# Antifouling and Antimicrobial Study of Nanostructured Mixed-Matrix Membranes for Arsenic Filtration

**DOI:** 10.3390/nano13040738

**Published:** 2023-02-15

**Authors:** Tawsif Siddique, Sheeana Gangadoo, Duy Quang Pham, Naba K. Dutta, Namita Roy Choudhury

**Affiliations:** 1 Chemical and Environmental Engineering, School of Engineering, RMIT University, Melbourne, VIC 3000, Australia; 2 College of Medicine and Public Health, Flinders University, Sturt Road, Bedford Park, SA 5042, Australia

**Keywords:** polysulfone, mixed-matrix membrane, arsenic, antifouling, antibacterial

## Abstract

Membrane fouling is a major drawback in the membrane filtration industry for water treatment. Mixed-matrix membranes (MMMs) are well known for their enhanced antifouling and antibacterial properties, which could offer potential benefits for membrane filtration processes in the water treatment field. In this work, three electrospun nanofibrous MMMs (P, CP, and MCP, which were, respectively, the pristine polysulfone membrane and mixed-matrix membranes (MMMs) consisting of GO–ZnO and GO–ZnO–iron oxides) were studied for antifouling and antibacterial properties with respect to the arsenic nanofiltration process. The effects of these composites on the antifouling behaviour of the membranes were studied by characterising the bovine serum albumin (BSA) protein adsorption on the membranes and subsequent analysis using microscopic (morphology via scanning electron microscopy) and Brunauer–Emmett–Teller (BET) analyses. The antibacterial properties of these membranes were also studied against Gram-positive *Staphylococcus aureus* (*S. aureus*) and Gram-negative *Escherichia coli* (*E. coli*). The composite nanoparticle-incorporated membranes showed improved antifouling properties in comparison with the pristine polysulfone (PSF) membrane. The excellent antimicrobial properties of these membranes make them appropriate candidates to contribute to or overcome biofouling issues in water or wastewater treatment applications.

## 1. Introduction

Water shortage and limited access to pure drinking water are among the major concerns of the current world. Unfortunately, a large portion of the world’s population is deprived of easy access to this basic human need. Although the Earth contains a large amount of water, only a little part is suitable for human consumption. Moreover, water sources are being polluted because of climate change, poor waste management, and environmental pollution. Some pollutants of water are microorganisms, microplastics, Perfluoroalkyl and Polyfluoroalkyl substances (PFAS), heavy metals, organic dye, and oxyanions of metals [1,2,3,4]. Arsenic oxyanion contamination is very serious, as it occurs as a result of natural phenomena and cannot be controlled. Arsenic consumption can cause many diseases and can even be lethal at high concentrations [5,6]. With the increasing demand for drinkable water, nontraditional water resources are being taken into consideration as a source of water by treating the water in various ways.

**Figure 1:**
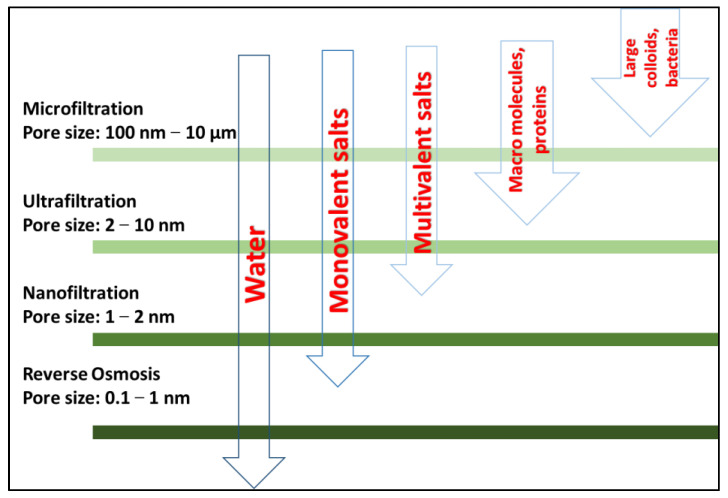
Type of pollutants removed by each class of membrane.

Water purification membranes can filter out wide ranges of contaminants of different sizes based on the type of membrane. The most commonly developed membrane separation processes are known as reverse osmosis (RO), electrodialysis (ED), microfiltration (MF), ultrafiltration (UF), and nanofiltration (NF). Microfiltration, ultrafiltration, nanofiltration, and reverse osmosis are pressure-driven processes, whereas electrodialysis is an electro-driven process [7]. Choosing the proper membrane type for a specific type or group of pollutants is crucial. In a pressure-driven porous membrane system, the separation process mainly follows the mechanism of size exclusion, i.e., separation is carried out based on the size of the contaminants present in water [8]. As shown in Figure 1, the most appropriate type of membrane for arsenic removal is a nanofiltration membrane due to its ability to eliminate the presence of multivalent ions in water [9].

Polymer membranes are widely used for membrane preparation because of their low cost, high selectivity, and easy film-forming capability. However, they suffer from low durability and fouling tendency [10]. Fouling is the unwanted deposition of solutes on the membrane surface and within the pores as well, which causes mass transfer resistance, low water flux, and low selectivity. Fouling can be caused by several phenomena such as the intrinsic hydrophobicity of the polymer, which favours the adherence of hydrophobic natural organic matters and deposition of foulants from wastewater and is categorised as organic fouling, inorganic fouling, and bio-colloidal fouling [11,12,13]. It results in crippled membrane performance, and pristine polymer membranes are unable to deal with this problem. The hydrophilisation of the polymer membrane is one of the most popular methods to mitigate this problem [14]. Fortunately, there is a well-established strategy for the introduction of nanoparticles in the polymer matrix for enhancing hydrophilicity. Therefore, mixed-matrix nanostructure membranes are widely being studied. Plenty of research studies have reported the antifouling and antibacterial effects of TiO_2_ and ZnO [15,16,17,18]. Moreover, with TiO_2_ being expensive, several reports proved that ZnO is the best alternative to TiO_2_, as ZnO is cheap in addition to having other desired properties such as antifouling and antibacterial properties and hydrophilicity [19,20]. However, the proper dispersion of zinc oxide on the polymer matrix without agglomeration is often challenging. Graphene oxide (GO) is favourable as a supporting sheet for proper dispersion in membrane fabrication [21,22]. The functional groups of GO and the synergistic effect between nanoparticles and hydrophilic layered GO contribute to the formation of hybrid nanostructured particles [23]. Ying et al. reported the application of GO–ZnO composite nanoparticles in polysulfone (PSF) membranes for better distribution with enhanced antifouling and antibacterial properties [24]. Studies also suggest the application of iron oxide in mixed-matrix membrane preparation for a considerable removal rate of arsenic, nontoxicity, antibacterial and antifouling properties. Several studies reported the antimicrobial property of Fe_3_O_4_ [25]. Lee et al. reported the application of iron-based nanoparticles for the deactivation of *E. coli* cells [26]. Iron oxide hybrids with other particles showed significant antimicrobial properties against ten bacterial strains and four *Candida* species [27,28]. Thus, it was speculated that the incorporation of iron oxide with GO–ZnO for making polysulfone-based mixed-matrix membranes would offer strong antibacterial and antifouling properties.

Therefore, an attempt was made to investigate the antibacterial and antifouling properties of PSF electrospun membranes and PSG modified with nanoparticles for arsenic filtration as a continuation of our previous study reported earlier [29].

## 2. Materials and Methods

### 2.1. Materials

All chemicals used in the experiments were of reagent grade. Sodium (meta)arsenite (90%) and sodium arsenate dibasic heptahydrate were obtained from Sigma-Aldrich and were used to prepare the arsenic-contaminated water. Bovine serum albumin (BSA, Sigma-Aldrich, St. Louis, MO, USA) was used to study the antifouling behaviour of the membranes. Deionised (DI) water was used in sample preparation and for pure water flux measurements. The Gram-positive bacterium methicillin-resistant *Staphylococcus aureus* (ATCC 6538) and the Gram-negative bacterium *Escherichia coli* (ATCC 25922) were used for the antibacterial study of the membranes.

Three electrospun nanofibrous mixed-matrix membranes were used for this study, as reported earlier [29]. The membranes were P, CP, and MCP. P was a pristine polysulfone (PSF) membrane, CP consisted of 1 wt% GO–ZnO composite particles in the PSF matrix, and MCP consisted of 1 wt% GO–ZnO–iron oxide composite particles in the PSF matrix.

### 2.2. Antifouling Study

Antifouling experiments were performed using crossflow filtration equipment as mentioned in our previous study [29] for pure water and arsenic-contaminated water at different levels of pressure between 2 bar and 7 bar. First, pure or arsenic-contaminated water was passed through the membrane for 80 min at operating pressure, resulting in a stable water flux (J_1_), which was recorded. Secondly, 1 mg/mL BSA solution was filtered for 80 min at the same pressure, and the protein solution flux (J_p_) was determined. Then, the membranes were simply washed with deionised water for 15 min, and then the pure water flux of cleaned membranes (J_2_) was measured again in the same way as mentioned in the first step. This was repeated three times for each pressure level for P, CP, and MCP, and the average values were used to calculate the flux recovery ratio (FRR), the total resistance (R_t_), the irreversible resistance (R_ir_), and the reversible resistance (R_r_) according to Equations (1)–(4), respectively, at different pressure levels.

FRR = J_2_/J_1_ (1)

R_t_ = 1 − (J_p_/J_1_) (2)

R_ir_ = (J_1_ − J_2_)/J_1_ (3)

R_r_ = (J_2_ − J_p_ )/J_1_ (4)

A higher FRR value represents the better antifouling property of the membrane, and R_t_ is the sum of R_ir_ and R_r_.

For further investigation of the mechanism of membrane fouling, Brunauer–Emmett–Teller analysis (BET; TriStar II 3020, Micromeritics Instrument Corporation, Australia) was carried out. An ultra-high resolution (UHR) scanning electron microscope (FEI Nova NanoSEM 200, FEI, Hillsboro, OR, USA) was used to observe the surface morphology of the fouled membranes, and a goniometer (OCA20, Particle and Surface Science Pty. Ltd., Gosford, NSW, Australia) was used to measure the contact angle of the static water to study the surface wettability nature of the membranes.

### 2.3. Antibacterial Test

The antibacterial behaviour of three membranes (P, CP, and MCP) was evaluated against the Gram-positive methicillin-resistant *S. aureus* (MRSA) and the Gram-negative *E. coli*. For qualitative observation, microbial solutions of MRSA and *E. coli* were prepared in phosphate-buffered saline (PBS) with an optical density (OD_600_) of ~0.1. A sterile cotton swab was dipped in the bacterial solution and used to streak the microbial solutions onto Luria–Bertani (LB) agar plates. To obtain uniform growth, the whole plate was streaked with the swab in one direction, rotated by 60°, and streaked again. The plate was allowed to dry for approximately 5 min, followed by gently placing membranes on inoculated agar plates using sterilised forceps. After incubating at 37 °C for 24 h, the plates were examined for bacterial growth. This procedure was performed separately for MRSA and *E. coli.*

Quantitative antibacterial behaviour was also observed using confocal microscopy. The P, CP, and MCP membranes were prepared again with microbial solutions of MRSA and *E. coli* at OD_600_ 0.1 using PBS and incubated for 24 h at 37 °C. Following incubation, the membranes were carefully washed with sterile PBS. The membranes were stained with 15–20 µL of a LIVE/DEAD BacLightTM Viability Kit (including SYTO 9 and propidium iodide) (Molecular ProbesTM, Invitrogen, Grand Island, NY, YSA) and incubated in the dark at room temperature for 10 mins, according to the manufacturer’s protocol. SYTO 9 dye is cell-permeable and can bind to nucleic acids of both live and dead cells, while PI dye can only enter cells with damaged membranes, as it is nonpermeable and replaces SYTO 9 and binds to the nucleic acids due to its stronger affinity [30]. Following incubation with the dye, the membranes were washed twice more with sterile PBS and placed in a fluorodish containing sterile PBS. The visualisation of the membranes’ antimicrobial efficacy was investigated using a ZEISS LSM 880 Airyscan upright microscope (Zeiss, Oberkochen, Germany) and the proportion of live-to-dead cells was analysed using ImageJ software (U.S. National Institutes of Health, Bethesda, MD, USA).

## 3. Results and Discussion

### 3.1. Antifouling Behaviour of Nanostructured Mixed-Matrix Membranes

**Figure 2:**
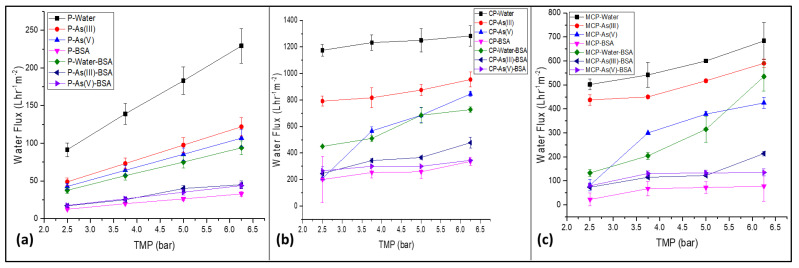
Water flux of the membranes (**a**) P, (**b**) CP, and (**c**) MCP as a function of TMP for pure and contaminated water during BSA filtration for antifouling study.

Filtration fouling tests were carried out to investigate and study the antifouling properties of the pristine and mixed-matrix PSF membranes. BSA was used as a protein model in this study. Figure 2 shows the change in the water flux behaviour of the three different membranes in terms of water contamination at different transmembrane pressure (TMP) values between 2.5 and 6.25 bars. The water flux at a specific TMP value was the highest in the case of pure water, whereas the lowest was for BSA-contaminated water. All three membranes (P, CP, and MCP) showed the same trend. Membrane fouling resulted in a decrease in water flux in all the membranes for pure water and contaminated water, which is clearly shown in Figure 2. Similar trends were reported in previous studies [31,32]. As shown in Figure 2, the antifouling behaviour of the membranes was determined, and FRR, R_t_, R_ir,_ and R_r_ were calculated using Equations (1)–(4), respectively.

**Figure 3:**
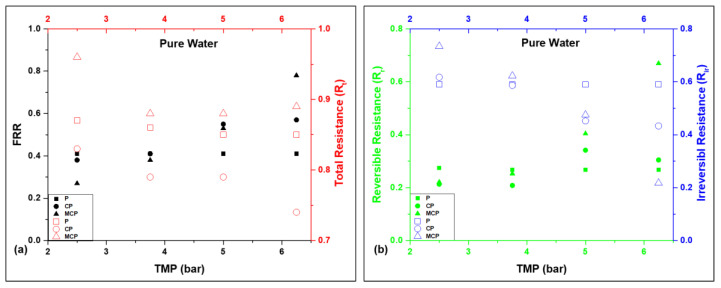

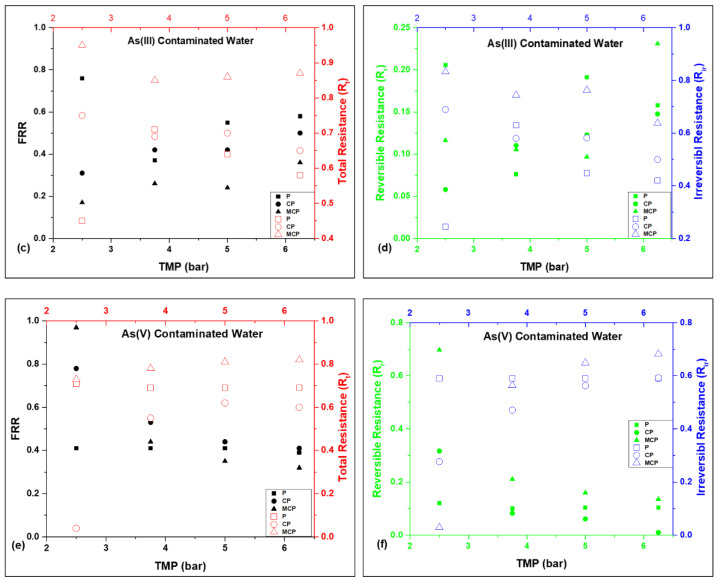
(**a**,**c**,**e**) Fouling recovery ratio and total fouling resistance of the membranes as a function of TMP in terms of pure and arsenic-contaminated water, and (**b**,**d**,**f**) reversible and irreversible resistance of the membranes as a function of TMP in terms of pure and arsenic-contaminated water.

Figure 3a shows the results of the flux recovery ratio (FRR) and the total resistance (R_t_) for the pristine PSF and mixed-matrix PSF membranes as a function of pressure for pure water. No significant pressure effect was observed on the FRR of the pristine PSF membrane (P) for pure water, as it was reported to be constantly 0.41 with the pressure change from 2.5 to 6.25 bars, whereas the FRR value increased with the increase in pressure for CP and MCP. The mixed-matrix PSF membranes showed a higher % FRR than the pristine PSF membranes at 6.25 bars due to the presence of composite particles on the PSF matrix, as ZnO and GO enhance the antifouling activity of membranes [32,33,34]. Between the two mixed-matrix PSF membranes CP and MCP, MCP had an FRR of 0.78, which was higher than CP (0.57) at 6.25 bar due to the presence of iron oxide in the composite particles in addition to GO and ZnO; iron oxide enhanced the membrane’s hydrophilicity and antifouling activity [35]. The lower FRR of MCP, compared with CP, at a lower pressure value could be due to CP having a higher water flux than MCP at lower pressure levels. On the other hand, the total fouling resistance was not much affected by TMP (Figure 3a), as the change was only up to 0.09 in R_t_ for the pressure change of 3.75 bars, whereas the fouling resistance decreased with the pressure increase for all the membranes. MCP showed the highest resistance to fouling (0.88–0.96), indicating better antifouling properties among the three PSF membranes, as MCP consisted of composite GO, ZnO, and iron oxide particles.

An antifouling study was carried out with As(III)- and As(V)-contaminated water to understand the membranes’ antifouling behaviour specifically for arsenic filtration. Figure 3c shows the results of the flux recovery ratio (FRR) for the pristine PSF and mixed-matrix PSF membranes as a function of pressure for As(III)-contaminated water. In the case of the pristine PSF membrane (P), the FRR decreased by 51.3% when the pressure increased by 1.25 bar, which is an unfavourable membrane property for nanofiltration. In contrast, the FRR was directly proportional to the applied transmembrane pressure for the mixed-matrix membranes CP and MCP; a lower interaction time between the membrane surface and water at a higher pressure could be a reason for this. CP had higher fouling ratios than MCP due to the interaction between the As(III) and membrane surface, where GO, ZnO, and iron oxide contributed to removing arsenic from water and to its adsorption. Similarly, the FRR as a function of TMP was studied for As(V) (Figure 3e). The FRR of the pristine PSF membrane was not much affected by pressure, as observed for pure water, whereas the fouling ratio of the mixed-matrix membranes decreased with the increase in the applied transmembrane pressure. In this study, MCP had higher fouling ratios than CP for As(V)-contaminated water, in contrast to the determined values for As(III)-contaminated water. The reason for this could be the interaction between As(V) and the nanoparticles present in the membrane’s fibre matrix.

The total fouling resistance of the membranes for As(III) was inversely proportional to the TMP for the pristine and mixed-matrix PSF membranes (Figure 3c). Although the pristine PSF membrane was much more affected by TMP than the mixed-matrix membranes, the changes in the R_t_ of CP and MCP were only 0.1 and 0.08, respectively, with the change in pressure between 2.5 and 6.25 bar. The mixed-matrix PSF membrane with GO–ZnO–iron oxide particles had the highest resistance to fouling (0.85–0.95) compared with P and CP. This trend was similar to that of pure water. This represents the better antifouling property of MCP among the three membranes studied, as it consisted of GO, ZnO, and iron oxide. The R_t_ of the pristine PSF membrane in the case of As(V)-contaminated water followed the same decreasing trend with the pressure increase, although marginally, whereas R_t_ was directly proportional to TMP for CP and MCP (Figure 3e). MCP had the highest total fouling resistance for pure and As contaminated water, which indicated that among the studied membranes, MCP had better antifouling behaviour in pure and As contaminated water.

There are two types of membrane fouling, which are hydraulically reversible resistance (R_r_) and hydraulically irreversible resistance (R_ir_). Weakly attached foulants (i.e., protein) on the membrane surface are known as reversible resistance, which can be dislodged through physical and chemical cleaning, whereas the strong adherence of foulants to the membrane results in irreversible fouling and cannot be removed through physical and chemical cleaning. Figure 3b,d,f show the results of R_r_ and R_ir_ as a function of TMP for the membranes, and the reported R_t_ in Figure 3a,c,e is the total of R_r_ and R_ir_. As for the FRR, there was also no effect on R_r_ and R_ir_ in the case of the pristine PSF membrane, as it resulted in 0.27 and 0.59, respectively, with the change in pressure from 2.5 to 6.25 bars for pure water, whereas it followed a different trend for arsenic-contaminated water. Moreover, the pressure had a noticeable effect on the reversible and irreversible resistances of the mixed-matrix membranes CP and MCP for pure and contaminated water. In the case of the mixed-matrix membranes, R_r_ increased with the increase in pressure, whereas R_ir_ was inversely proportional to TMP, as the combination of high pressure and lower porosity results in higher reversible resistance [33]. The R_r_/R_t_ ratio was higher than R_ir_/R_t_ for the mixed-matrix membranes, compared with the pristine PSF membrane, which indicates that the BSA protein adsorbed onto the top surface of these membranes can be easily washed off during the cleaning process. Therefore, better membrane performance was achieved.

To summarise, the flux recovery ratio (FRR), irreversible fouling (R_ir_), and reversible fouling (R_r_) of the composite nanoparticle-embedded membranes were enhanced, indicating that the surface properties of the membranes significantly contributed to the final membrane properties. 

#### 3.1.1. Membrane Surface Morphology and Contact Angle

**Figure 4:**
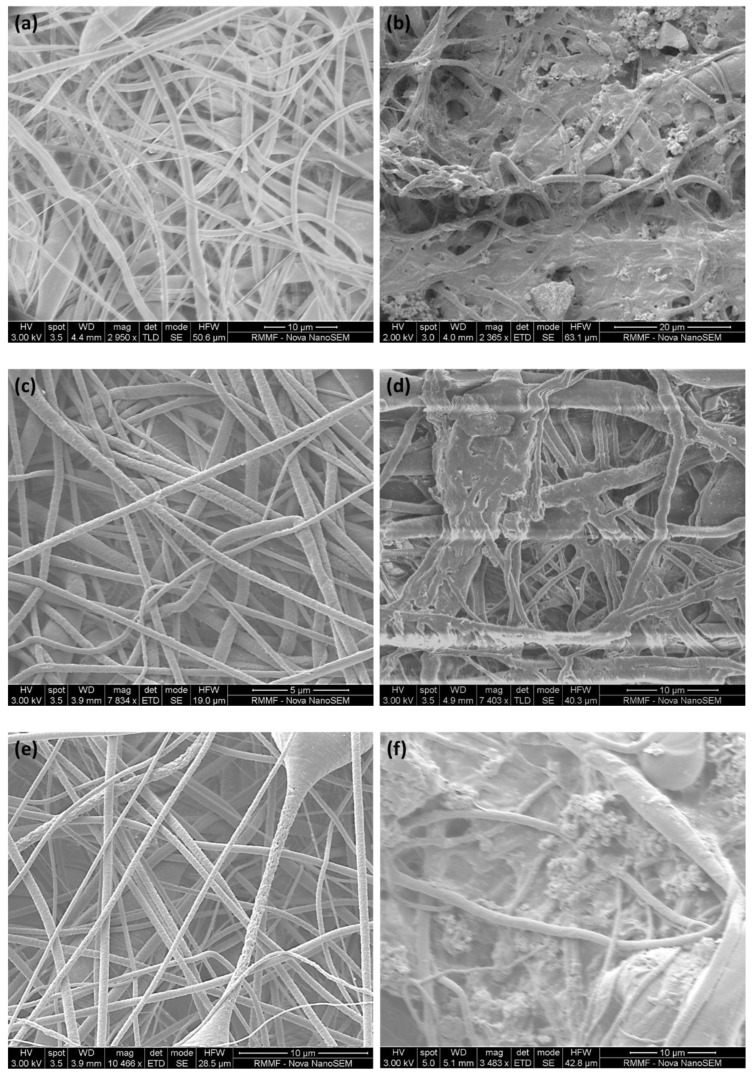
Morphology of the membrane before (**a**,**c**,**e**) and after (**b**,**d**,**f**) BSA filtration for antifouling study: (**a**,**b**) P, (**c**,**d**) CP, and (**e**,**f**) MCP.

Figure 4 illustrates the surface morphology of the as-fabricated and BSA-fouled membranes. As seen from comparing the images of the fouled and as-fabricated membranes, a fouling layer was formed on different areas of the membrane surface, and some pores were blocked by BSA, which resulted in the fouling recovery ratio and fouling resistance mentioned earlier. This surface change and pore blockage also resulted in the change in the water’s contact angle reported in Table 1. The BSA-fouled P and MCP membranes did not show any contact angle value, as the water was absorbed by the fouled membranes, whereas the fouled CP membrane showed a water contact angle of 56° due to the deposition of BSA on the membrane surface and the blockage of the pores, resulting in more than 50% decrease in pore volume in the case of P and MCP, compared with 33% in the case of CP (Table 1).

#### 3.1.2. Membrane Fouling Mechanism Study via BET

**Figure 5:**
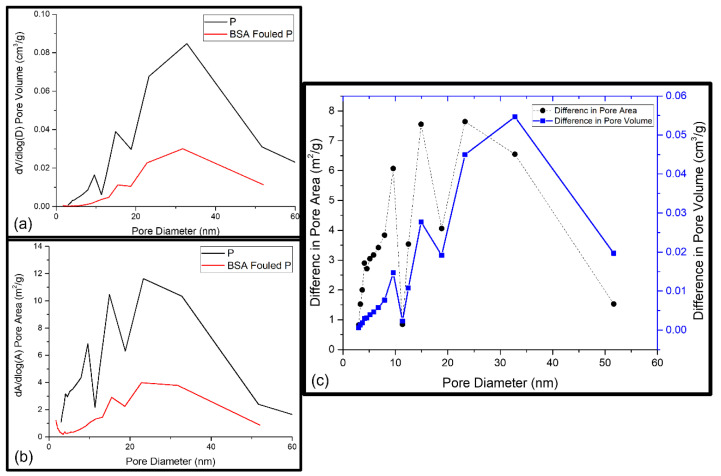
(**a**) BET pore area distribution of P and BSA-fouled P membranes; (**b**) BET pore volume distribution of P and BSA-fouled P membranes; (**c**) the difference between the pore area of P and BSA-fouled P and the difference between the pore volume of P and BSA-fouled P as a function of pore diameter.

**Figure 6:**
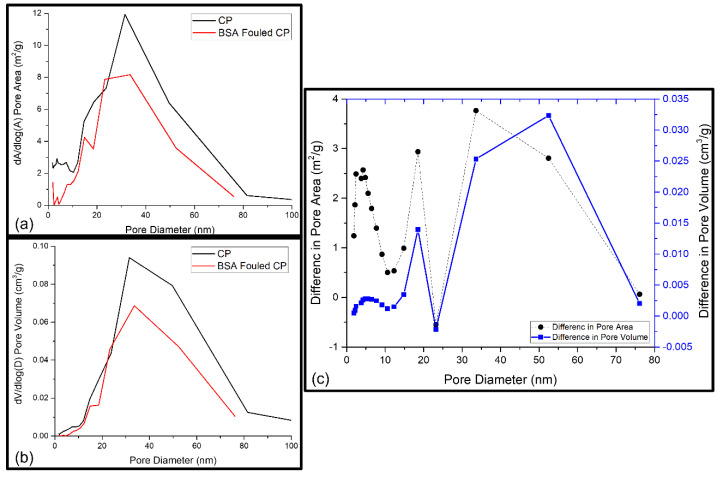
(**a**) BET pore area distribution of CP and BSA-fouled CP membranes; (**b**) BET pore volume distribution of CP and BSA-fouled CP membranes; (**c**) the difference between the pore area of CP and BSA-fouled CP and the difference between the pore volume of CP and BSA-fouled CP as a function of pore diameter.

**Figure 7:**
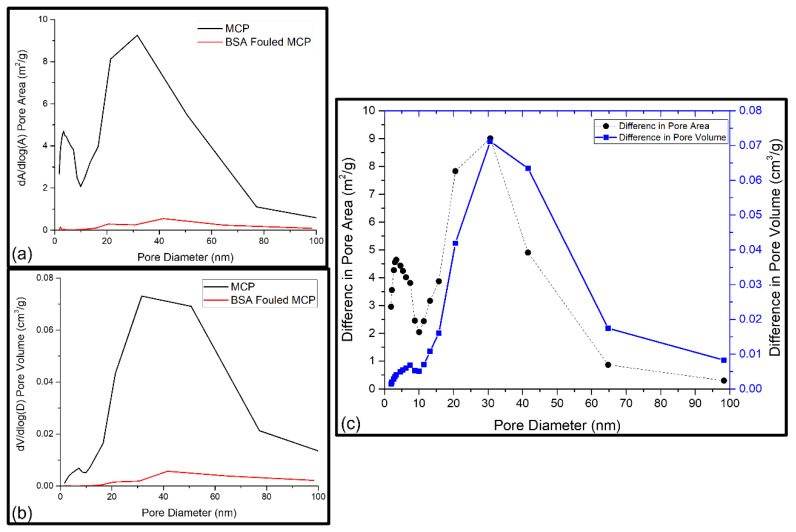
(**a**) BET pore area distribution of MCP and BSA-fouled MCP membranes; (**b**) BET pore volume distribution of MCP and BSA-fouled MCP membranes; (**c**) the difference between the pore area of MCP and BSA-fouled MCP and the difference between the pore volume of MCP and BSA-fouled MCP as a function of pore diameter.

The synthesised PSF membranes used in this study had an asymmetric structure, consisting of different layers of nanofibers. Due to this effect, the pores from all layers as well as the pores on fibre surfaces were included in the studied membrane samples, and the pore volumes of the layers and fibre surface together indicated the total pore volume. A possible foulant layer might be porous as well. Membranes are mesoporous and have pore sizes ranging from 0 to 100 nm [29]. Thus, the fouling-induced changes in the micropore region (<2 nm), mesopore region (2–50 nm), and macropore region (≥50 nm) were of interest. Further information about the porosity as a function of pore diameters or as a total pore volume in the whole-pore size distribution was revealed from BET analysis. Table 1 shows the acquired cumulative BET surface area and the cumulative pore surface area, volume, average desorption pore size, and fraction of micropores. As these cumulative or average values did not reveal any information about the pores responsible for the changes that occurred, the pore volume and pore area distributions of the reference and BSA-fouled membranes were also determined, which are presented in Figure 5, Figure 6 and Figure 7. The differences in the porosity distributions were also compared between the prepared and BSA-fouled membranes in terms of both pore area and pore volume.

##### Fouling of Pristine PSF membrane (P)

All the membranes were examined and compared for water flux, rejection, and water contact angle study. The pure PSF membrane was used for comparison. Porosity and surface area are considered the key parameters of the membrane’s structural properties to identify the sites with possible foulant accumulation. An increase of 11.4% in the BET surface area and a decrease of 64% in the cumulative pore surface area and 69% in the cumulative pore volume were found for the P membrane after fouling with BSA (Table 1). However, the average desorption pore size revealed a decrease of 13%, which probably resulted from the blockage of the widest pores of the membrane [36]. Figure 5a,b show the pore area and pore volume distributions of the reference and fouled membranes. Because the number of pores present at a certain pore diameter determined the surface area at that particular pore diameter, a decrease in pore volume at a certain pore width occurred with a decrease in the pore area at the same pore width (see Figure 5c). Correspondingly, an increase in pore volume at a certain pore width occurred with an increase in the pore area at the chosen pore width [36]. This can be seen in Figure 5c when comparing the pore volume and pore area distributions. When comparing the distributions of the reference membrane with the fouled one, it can be seen that the pores with diameters up to 25 nm underwent a small increase in pore volume but a relatively great increase in pore area. The nature of the interaction between the membrane and the foulant determines the extent of the adsorption of foulants on the surface and the pore walls of the membrane. When the interactions are favourable, adsorptive fouling takes place, and this could lead to cake-layer build-up [36]. In contrast to that, a sharp increase in pore volume was observed with a sharp decline in pore area between the pore size of 25 and 35 nm. The reason could be the filling of bigger pores with larger BSA molecules [36]. BSA fouling during nanofiltration is believed to occur through two distinct mechanisms: (1) the physical deposition of large protein aggregates on or within the membrane structure, and (2) the chemical attachment of native (nonaggregated) BSA to these previously deposited aggregates. Native BSA can only foul the membrane through chemical attachment to an existing protein deposit via the formation of intermolecular disulphide linkages.

##### Fouling of Mixed-Matrix PSF Membrane with GO–ZnO Composite Particles (CP)

The blockage of the wide pores of the membrane by BSA molecules causes a 10% decrease in the average desorption pore size (Table 1). However, more than a 50% increase in the BET surface area and a 39% and 33% decrease in, respectively, the cumulative pore surface area and volume were also observed. The pore area and pore volume distributions of the CP mixed-matrix membrane and the fouled CP membrane are shown in Figure 6a,b. The pore volume and pore area simultaneously increased and decreased at a certain pore width, as the surface area is calculated from the number of pores present at a certain pore diameter (Figure 6c) [36]. This can be clearly inferred from the comparison of pore volume and pore area distributions. When comparing the distributions of the conditioned membrane with the fouled one, it can be seen that the pores with diameters of 20 to 25 nm exhibited a reduced pore volume and area as a result of fouling. The wider pores may be filled with larger molecules, resulting in a decrease in the available pore volume and a decrease in the average pore size. In contrast to that, smaller pores (<20 nm) exhibited a small increase in pore volume but a relatively great increase in pore area, which originated from the BSA cake-layer build-up on the membrane surface [36]. A sharp increase in pore volume was observed with a sharp decline in pore area between the pore size of 35 and 55 nm, possibly due to the filling of bigger pores with larger BSA molecules [36]. Thus, both physical and chemical interactions governed protein fouling during the nanofiltration of GO–ZnO composite nanoparticles incorporated in the PSF mixed-matrix membranes.

##### Fouling of Mixed-Matrix PSF Membrane with GO–ZnO–Iron Oxide Composite Particles (MCP)

Table 1 shows the surface properties of the MCP membrane before and after BSA filtration. The BET surface area increased by 51%, whereas the pore surface area and pore surface volume decreased by 53% and 48%, respectively (Table 1). The average desorption pore size of MCP decreased by 45% after BSA fouling, which was the maximum amount in this study. This indicates that BSA molecules blocked the wide pores of MCP more than that of P and CP. Therefore, pore blockage was the main driving force during fouling for MCP. At a certain pore width, the pore area and pore volume were affected in the same manner because the reported pore area and volume were associated with the same number of pores at that specific pore width, as presented in Figure 7a,b [36]. Membrane fouling decreased the average pore diameter and increased the cumulative pore volume. To understand this finding, the pore volume and pore area distributions were compared, as shown in Figure 7c, by calculating and presenting the difference of pore area and pore volume between the reference and fouled MCP membranes as a function of pore diameter. The pores with diameters up to 100 nm exhibited a small increase in pore volume but a relatively large increase in pore area, which originated from the BSA layer formed on the MCP membrane’s surface due to both physical and chemical adsorption [36].

### 3.2. Antimicrobial Behaviour of Nanostructured Mixed-Matrix Membranes

**Figure 8:**
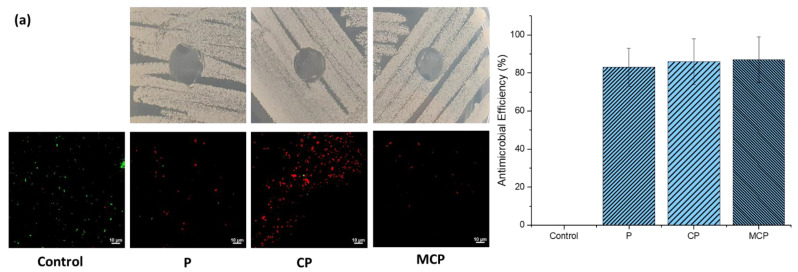

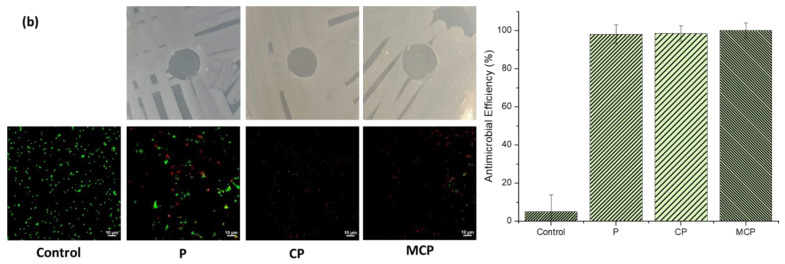
Antibacterial activity of P, CP, and MCP membranes for (**a**) MRSA and (**b**) *E. coli*.

Figure 8 reveals the results of the antibacterial tests of different membranes against MRSA (Figure 8a) and *E. coli* (Figure 8b). The pristine PSF membranes and composite nanoparticle-incorporated PSF membranes did not show any bacterial growth in their presence. It can, therefore, be concluded that these membranes have an antibacterial effect against *S. aureus* and *E. coli*.

The antibacterial behaviour of the membranes was further studied by performing a quantitative assessment of % dead cells using confocal microscopy, which was reported as antimicrobial efficiency. As shown in Figure 8, live cells are represented by high intensity of green fluorescence, and dead cells are represented by high intensity of red fluorescence. Figure 8a demonstrates the antibacterial efficacy (%) of P, CP, and MCP against Gram-positive MRSA with 83%, 86%, and 87%, respectively. This trend was similar for Gram-negative *E. coli*, as shown in Figure 8b, with values of 98%, 98.5%, and 100% for P, CP, and MCP, respectively. From this result, it can be concluded that the antibacterial activity increased with the addition of GO–ZnO and GO–ZnO–iron oxide composite nanoparticles due to the antibacterial properties of GO, ZnO, and iron oxide particles, separately as well as in combined form [24,37,38]. The antibacterial mechanism of these nanoparticles incorporated in the membranes can give a clear picture of this increased antibacterial behaviour. Antibacterial surfaces kill bacteria by releasing biocides, direct contact with cell membranes, or expressing cationic polymers. Through a contact-killing mechanism, bacteria can be inactivated due to the nanomaterial’s nature or geometry and the strong interactions induced between the nanomaterial and bacterial cells.

A detailed structural study of the bacteria will help to understand the antibacterial mechanism of the membranes. A bacterium is made of a cell membrane, cell wall, and cytoplasm, and the cell wall lies outside the cell membrane and is composed mostly of a homogeneous peptidoglycan layer (which consists of amino acids and sugars) and maintains the osmotic pressure of the cytoplasm and also the characteristic cell shape [19]. Gram-positive bacteria have one cytoplasmic membrane with a multilayer of peptidoglycan polymer and a thicker cell wall of 20–80 nm, but Gram-negative bacteria wall is composed of two cell membranes: an outer membrane and a plasma membrane with a thin layer of peptidoglycan of 7–8 nm [19,39]. Nanoparticles within this range can easily pass this peptidoglycan layer and damage the bacteria cell. Consequently, proteins, carbohydrates, nucleic acids, salts, ions, and almost 80% water are present in the cytoplasm and result in negatively charged bacterial cell walls [19]. Therefore, a negatively charged membrane surface inhibits bacterial growth through the repulsive force between the bacterial cell wall and the membrane surface [24], as the membrane surface charges were reported as negative at different pH values in our earlier studies [29].

GO is hydrophilic as well because it has unique surface tuning properties [24,37,40]. The hydrophilicity of the membranes also plays a role in the inhibition of bacterial growth [41], and the addition of GO increases the hydrophilicity of membranes, as per our previous study [29]. Another part of the composite particles, ZnO, is hydrophilic, nontoxic, and biocompatible, and it also shows good photocatalytic activity [37], whereas the direct contact of ZnO nanoparticles with bacteria cell walls causes the destruction of bacterial cell integrity [42,43,44] and the liberation of antimicrobial ions (Zn^2+^ ions) [45,46,47] and also releases hydrogen peroxide, hydroxide, and superoxide anion through ROS generation [48,49,50,51]. Therefore, the regrowth of bacteria cells is completely arrested in CP membranes due to the synergistic effects of GO–ZnO, resulting in the formation of ROS, including HO*, H_2_O_2_, and O_2_*, and the release of Zn^2+^ ions by enhancing the electron transfer [24,52,53]. In this study, MCP membranes showed the maximum antimicrobial efficiency, as the presence of iron oxide in the composite particles made the penetration of the nanoparticles into the bacteria cells easier and inactivated them, which resulted in more inhibition of bacterial growth [25,26,37,54].

The antimicrobial activity of the materials was greater towards the Gram-negative bacteria (*E. coli*) than towards the Gram-positive bacteria (MRSA), possibly due to the thinner layer of peptidoglycan present, as a result of which the material had a higher degree of contact and penetration with the membrane. The studies investigating the antimicrobial activity of similar graphene oxide composites support these findings [55,56]. The increase in the dead-cell proportion of MRSA occurred with the inclusion of ZnO and ZnO iron oxides, where the mechanism of killing action could be mainly attributed to the release of reactive oxygen species (ROS) and Zn^2+^ ions rather than physical contact, as seen in Gram-negative *E. coli*. This observation highlights the significance of this composite material’s ability to target different types of bacteria and their cell membranes.

## 4. Conclusions

Mixed-matrix PSF membranes were prepared via the electrospinning method using GO–ZnO and GO–ZnO–iron oxide as filler particles. The composite nanoparticles improved the antifouling properties of mixed-matrix PSF membranes, compared with the pristine PSF membranes, by improving the FRR ratio. Due to the added composite particles, the ratio of R_r_/R_t_ increased. This indicated that the BSA molecules adsorbed onto the top surface of mixed-matrix PSF membranes were more easily washed off during cleaning. The BSA layer on top of the membrane surface and the BSA molecule’s adsorption into the membrane pores of different sizes led to BSA fouling, as seen from BET analysis. Additionally, the antibacterial activity also increased with the addition of the composite particles. These results are of particular importance to the arsenic filtration industry since improved antifouling and flux recovery can help reduce operating and maintenance costs in the filtration processes using these membranes, and antibacterial behaviour can hinder bacterial growth, thus ensuring high-quality water.

## Figures and Tables

**Table 1:** Surface properties of the membranes before and after BSA filtration.

Membranes		Contact Angle (°)	BET Surface Area (m^2^/g)	Pore Surface Area (m^2^/g)	Pore Volume (cm^3^/g)	Desorption Pore Size (nm)	Micropore Area (m^2^/g)	Micropore Volume (cm^3^/g)
P	Before Fouling	125 ± 2	55.1	82.53	0.35	16.75	*	*
	BSA-Fouled Membrane	-	61.36	29.82	0.11	14.7	1.39	0.0003
CP	Before Fouling	104 ± 3	19.96	62.3	0.33	17.5	*	*
	BSA-Fouled Membrane	56 ± 3	40.89	38.13	0.22	15.72	0.19	*
MCP	Before Fouling	87 ± 5	18.97	76.07	0.31	25.11	*	*
	BSA-Fouled Membrane	-	38.37	35.61	0.16	13.7	*	*

* Fractions of the micropores were too low to be determined.

## Data Availability

The datasets generated for this study are available on request to the corresponding author.

## References

[1] Mastropietro T.F., Bruno R., Pardo E., Armentano D. (2021). Reverse osmosis and nanofiltration membranes for highly efficient PFASs removal: Overview, challenges and future perspectives. Dalton Trans..

[2] Poerio T., Piacentini E., Mazzei R. (2019). Membrane processes for microplastic removal. Molecules.

[3] Qdais H.A., Moussa H. (2004). Removal of heavy metals from wastewater by membrane processes: A comparative study. Desalination.

[4] Singh S., Sharma R., Khanuja M. (2018). A review and recent developments on strategies to improve the photocatalytic elimination of organic dye pollutants by BiOX (X = Cl, Br, I, F) nanostructures. Korean J. Chem. Eng..

[5] Shankar S., Shanker U. (2014). Arsenic contamination of groundwater: A review of sources, prevalence, health risks, and strategies for mitigation. Sci. World J..

[6] Shaji E., Santosh M., Sarath K.V., Prakash P., Deepchand V., Divya B.V. (2021). Arsenic contamination of groundwater: A global synopsis with focus on the Indian Peninsula. Geosci. Front..

[7] Baker R.W. (2000). Membrane technology. Kirk-Othmer Encyclopedia of Chemical Technology.

[8] Baker R.W. (2012). Membrane Technology and Applications.

[9] Yang Z., Zhou Y., Feng Z., Rui X., Zhang T., Zhang Z. (2019). A review on reverse osmosis and nanofiltration membranes for water purification. Polymers.

[10] Pendergast M.M., Hoek E.M. (2011). A review of water treatment membrane nanotechnologies. Energy Environ. Sci..

[11] Miller D.J., Dreyer D.R., Bielawski C.W., Paul D.R., Freeman B.D. (2017). Surface modification of water purification membranes. Angew. Chem. Int. Ed..

[12] Shirasaki N., Matsushita T., Matsui Y., Ohno K. (2008). Effects of reversible and irreversible membrane fouling on virus removal by a coagulation–microfiltration system. J. Water Supply Res. Technol. AQUA.

[13] Lin H., Peng W., Zhang M., Chen J., Hong H., Zhang Y. (2013). A review on anaerobic membrane bioreactors: Applications, membrane fouling and future perspectives. Desalination.

[14] Zhao C., Xue J., Ran F., Sun S. (2013). Modification of polyethersulfone membranes–A review of methods. Prog. Mater Sci..

[15] Kwak S.-Y., Kim S.H., Kim S.S. (2001). Hybrid organic/inorganic reverse osmosis (RO) membrane for bactericidal anti-fouling. 1. Preparation and characterization of TiO_2_ nanoparticle self-assembled aromatic polyamide thin-film-composite (TFC) membrane. Environ. Sci. Technol..

[16] Leong S., Razmjou A., Wang K., Hapgood K., Zhang X., Wang H. (2014). TiO_2_ based photocatalytic membranes: A review. J. Membr. Sci..

[17] Bet-Moushoul E., Mansourpanah Y., Farhadi K., Tabatabaei M. (2016). TiO_2_ nanocomposite based polymeric membranes: A review on performance improvement for various applications in chemical engineering processes. Chem. Eng. J..

[18] Zhao S., Yan W., Shi M., Wang Z., Wang J., Wang S. (2015). Improving permeability and antifouling performance of polyethersulfone ultrafiltration membrane by incorporation of ZnO-DMF dispersion containing nano-ZnO and polyvinylpyrrolidone. J. Membr. Sci..

[19] Sirelkhatim A., Mahmud S., Seeni A., Kaus N.H.M., Ann L.C., Bakhori S.K.M., Hasan H., Mohamad D. (2015). Review on Zinc Oxide Nanoparticles: Antibacterial Activity and Toxicity Mechanism. Nanomicro. Lett..

[20] Balta S., Sotto A., Luis P., Benea L., Van der Bruggen B., Kim J. (2012). A new outlook on membrane enhancement with nanoparticles: The alternative of ZnO. J. Membr. Sci..

[21] Bao Q., Zhang D., Qi P. (2011). Synthesis and characterization of silver nanoparticle and graphene oxide nanosheet composites as a bactericidal agent for water disinfection. J. Colloid. Interface Sci..

[22] Song J.J., Huang Y., Nam S.-W., Yu M., Heo J., Her N., Flora J.R., Yoon Y. (2015). Ultrathin graphene oxide membranes for the removal of humic acid. Sep. Purif. Technol..

[23] Wang Z., Yu H., Xia J., Zhang F., Li F., Xia Y., Li Y. (2012). Novel GO-blended PVDF ultrafiltration membranes. Desalination.

[24] Chung Y.T., Mahmoudi E., Mohammad A.W., Benamor A., Johnson D., Hilal N. (2017). Development of polysulfone-nanohybrid membranes using ZnO-GO composite for enhanced antifouling and antibacterial control. Desalination.

[25] Mukherjee M., De S. (2015). Reduction of microbial contamination from drinking water using an iron oxide nanoparticle-impregnated ultrafiltration mixed matrix membrane: Preparation, characterization and antimicrobial properties. Environ. Sci. Water Res. Technol..

[26] Lee C., Kim J.Y., Lee W.I., Nelson K.L., Yoon J., Sedlak D.L. (2008). Bactericidal Effect of Zero-Valent Iron Nanoparticles on *Escherichia coli*. Environ. Sci. Technol..

[27] Dallas P., Tucek J., Jancik D., Kolar M., Panacek A., Zboril R. (2010). Magnetically controllable silver nanocomposite with multifunctional phosphotriazine matrix and high antimicrobial activity. Adv. Funct. Mater..

[28] Prucek R., Tuček J., Kilianová M., Panáček A., Kvítek L., Filip J., Kolář M., Tománková K., Zbořil R. (2011). The targeted antibacterial and antifungal properties of magnetic nanocomposite of iron oxide and silver nanoparticles. Biomaterials.

[29] Siddique T., Balu R., Mata J., Dutta N.K., Roy Choudhury N. (2022). Electrospun Composite Nanofiltration Membranes for Arsenic Removal. Polymers.

[30] Buysschaert B., Byloos B., Leys N., Van Houdt R., Boon N. (2016). Reevaluating multicolor flow cytometry to assess microbial viability. Appl. Microbiol. Biotechnol..

[31] Nabe A., Staude E., Belfort G. (1997). Surface modification of polysulfone ultrafiltration membranes and fouling by BSA solutions. J. Membr. Sci..

[32] Zambare R.S., Dhopte K.B., Nemade P.R., Tang C.Y. (2020). Effect of oxidation degree of GO nanosheets on microstructure and performance of polysulfone-GO mixed matrix membranes. Sep. Purif. Technol..

[33] Wu H., Tang B., Wu P. (2014). Development of novel SiO_2_–GO nanohybrid/polysulfone membrane with enhanced performance. J. Membr. Sci..

[34] Shen L., Huang Z., Liu Y., Li R., Xu Y., Jakaj G., Lin H. (2020). Polymeric membranes incorporated with ZnO nanoparticles for membrane fouling mitigation: A brief review. Front. Chem..

[35] Said N., Hasbullah H., Abidin M.N.Z., Ismail A.F., Goh P.S., Othman M.H.D., Kadir S.H.S.A., Kamal F., Abdullah M.S., Ng B.C. (2019). Facile modification of polysulfone hollow-fiber membranes via the incorporation of well-dispersed iron oxide nanoparticles for protein purification. J. Appl. Polym. Sci..

[36] Virtanen T., Rudolph G., Lopatina A., Al-Rudainy B., Schagerlöf H., Puro L., Kallioinen M., Lipnizki F. (2020). Analysis of membrane fouling by Brunauer-Emmet-Teller nitrogen adsorption/desorption technique. Sci. Rep..

[37] Mukherjee M., De S. (2018). Antibacterial polymeric membranes: A short review. Environ. Sci. Water Res. Technol..

[38] Aryanti P., Sianipar M., Zunita M., Wenten I. (2017). Modified membrane with antibacterial properties. Membr. Water Treat..

[39] Fu G., Vary P.S., Lin C.-T. (2005). Anatase TiO_2_ nanocomposites for antimicrobial coatings. J. Phys. Chem. B.

[40] Zeng Z., Yu D., He Z., Liu J., Xiao F.-X., Zhang Y., Wang R., Bhattacharyya D., Tan T.T.Y. (2016). Graphene Oxide Quantum Dots Covalently Functionalized PVDF Membrane with Significantly-Enhanced Bactericidal and Antibiofouling Performances. Sci. Rep..

[41] Kochkodan V., Hilal N., Goncharuk V., Al-Khatib L., Levadna T. (2006). Effect of the surface modification of polymer membranes on their microbiological fouling. Colloid J..

[42] Brayner R., Ferrari-Iliou R., Brivois N., Djediat S., Benedetti M.F., Fiévet F. (2006). Toxicological impact studies based on *Escherichia coli* bacteria in ultrafine ZnO nanoparticles colloidal medium. Nano Lett..

[43] Zhang L., Jiang Y., Ding Y., Povey M., York D. (2007). Investigation into the antibacterial behaviour of suspensions of ZnO nanoparticles (ZnO nanofluids). J. Nanopart. Res..

[44] Adams L.K., Lyon D.Y., Alvarez P.J. (2006). Comparative eco-toxicity of nanoscale TiO_2_, SiO_2_, and ZnO water suspensions. Water Res..

[45] Kasemets K., Ivask A., Dubourguier H.-C., Kahru A. (2009). Toxicity of nanoparticles of ZnO, CuO and TiO_2_ to yeast Saccharomyces cerevisiae. Toxicol. Vitro.

[46] Brunner T.J., Wick P., Manser P., Spohn P., Grass R.N., Limbach L.K., Bruinink A., Stark W.J. (2006). In vitro cytotoxicity of oxide nanoparticles: Comparison to asbestos, silica, and the effect of particle solubility. Environ. Sci. Technol..

[47] Li M., Zhu L., Lin D. (2011). Toxicity of ZnO nanoparticles to Escherichia coli: Mechanism and the influence of medium components. Environ. Sci. Technol..

[48] Sawai J., Shoji S., Igarashi H., Hashimoto A., Kokugan T., Shimizu M., Kojima H. (1998). Hydrogen peroxide as an antibacterial factor in zinc oxide powder slurry. J. Ferment. Bioeng..

[49] Lipovsky A., Nitzan Y., Gedanken A., Lubart R. (2011). Antifungal activity of ZnO nanoparticles—The role of ROS mediated cell injury. Nanotechnology.

[50] Zhang L., Ding Y., Povey M., York D. (2008). ZnO nanofluids–A potential antibacterial agent. Prog. Nat. Sci..

[51] Jalal R., Goharshadi E.K., Abareshi M., Moosavi M., Yousefi A., Nancarrow P. (2010). ZnO nanofluids: Green synthesis, characterization, and antibacterial activity. Mater. Chem. Phys..

[52] Al-Hinai M.H., Sathe P., Al-Abri M.Z., Dobretsov S., Al-Hinai A.T., Dutta J. (2017). Antimicrobial Activity Enhancement of Poly(ether sulfone) Membranes by in Situ Growth of ZnO Nanorods. ACS Omega.

[53] Javdaneh S., Mehrnia M.R., Homayoonfal M. (2016). Engineering design of a biofilm formed on a pH-sensitive ZnO/PSf nanocomposite membrane with antibacterial properties. RSC Adv..

[54] Stanicki D., Boutry S., Laurent S., Wacheul L., Nicolas E., Crombez D., Vander Elst L., Lafontaine D.L.J., Muller R.N. (2014). Carboxy-silane coated iron oxide nanoparticles: A convenient platform for cellular and small animal imaging. J. Mater. Chem. B.

[55] El-Shafai N., El-Khouly M.E., El-Kemary M., Ramadan M., Eldesoukey I., Masoud M. (2019). Graphene oxide decorated with zinc oxide nanoflower, silver and titanium dioxide nanoparticles: Fabrication, characterization, DNA interaction, and antibacterial activity. RSC Adv..

[56] Archana S., Kumar K.Y., Jayanna B., Olivera S., Anand A., Prashanth M., Muralidhara H. (2018). Versatile graphene oxide decorated by star shaped zinc oxide nanocomposites with superior adsorption capacity and antimicrobial activity. J. Sci. Adv. Mater. Dev..

